# Correspondence on “Mass Balance Tracing of *In Vivo* Biodistribution, Relocation, and Excretion of Europium-Doped Micro/Nanoplastics in Rats”

**DOI:** 10.1021/acs.est.6c03540

**Published:** 2026-07-14

**Authors:** Alfonso M. Gañán-Calvo

**Affiliations:** Department of Aerospace Engineering and Fluid Mechanics, 16778 Universidad de Sevilla, 41092 Sevilla, Spain; ENGREEN, Laboratory of Engineering for Energy and Environmental Sustainability, Universidad de Sevilla, 41092 Sevilla, Spain

Luo and co-workers report an important quantitative study of intravenously administered europium-doped polystyrene micro/nanoplastics (MNPs) in rats and conclude that the level of brain accumulation was insignificant and among the lowest observed across examined tissues.^1^ They further cite our earlier preprint in connection with the suggestion that small-vascular-volume organs, such as the brain, may undergo selective bioaccumulation.^2^ This point is worth clarifying.

Our updated analysis, in a revised version posted in January 2026, no longer interprets vascularity itself as the mechanistic driver of chronic organ enrichment. Rather, vascular volume appears to be a collinear tissue descriptor that can correlate with but should not be conflated with the more plausible causal determinant, slow retention kinetics in lipid-rich parenchyma.^2^ The earlier vascularity-based wording is therefore better understood as a heuristic association rather than as a causal claim.

This distinction matters, because Luo et al. use a short-horizon intravenous kinetic experiment to argue against differential long-term brain accumulation. Their data do support limited early penetration of circulating particles into rat brain after a single intravenous administration,^1^ but that result does not resolve the separate question of whether, under chronic environmental exposure and very long residence times, the human brain can become disproportionately enriched relative to other organs.

The term “long term” is therefore being used at two very different temporal scales. In Luo et al., it refers to follow-up over three months after bolus intravenous exposure in rats, appropriately so, in contrast to shorter acute exposure designs.^1^ In contrast, the human organ data most relevant to public-health concerns reflect cumulative exposure and retention over many years, plausibly decades.^3^ These are not interchangeable regimes. A low brain burden at early or intermediate times in a small mammal does not exclude substantially higher asymptotic burdens in a large, lipid-rich organ subjected to lifelong exposure. In other words, a 90-day rat experiment is best interpreted as a preasymptotic kinetic snapshot, not as a direct proxy for chronic human steady state.

A second limitation is translational. The intravenous bolus design of Luo et al. is highly informative for mass balance pharmacokinetics, but it bypasses gastrointestinal uptake, trophic transfer, inhalation, and prolonged low-dose exposure, which are more relevant to chronic human accumulation scenarios.^1,2^ This distinction is not merely procedural. The route and dosing patterns can condition particle translocation, residence times, and organ partitioning. Intravenous rat biodistribution should therefore not be treated as a decisive test of hypotheses about lifelong human organ burdens.

Recent human data are directly relevant to this study. Nihart et al. reported frontal-cortex MNP concentrations that substantially exceeded those measured in the liver or kidney.^3^ The absolute values in that study have been debated on methodological grounds, especially with respect to contamination control and potential analytical interference,^4^ and the authors have replied in defense of their workflow and interpretation.^5^ However, the existence of that debate cuts against any definitive claim that the brain does not bioaccumulate differentially in the long term. Currently available human evidence is, at minimum, not conclusive on this question, and continued methodological improvement in the detection and quantification of MNPs in human tissues remains a priority for the field.^6,7^

Our retention-based framework reconciles these observations. Short-term kinetics may still be dominated by circulation, endothelial transfer, and reticuloendothelial sequestration, yielding weak early brain signals in the experimental animals. Over much longer times, however, organ-specific burdens can become controlled by tissue composition and clearance inefficiency, particularly in lipid-rich compartments. In that context, an apparent association with a small vascular volume can emerge without vascularity being the true causal driver. This is precisely why our revised preprint moved away from the earlier heuristic interpretation and toward a lipid-associated retention explanation.^2^

The conclusions of Luo et al. should therefore be interpreted more narrowly. Their study provides valuable evidence that a single intravenous dose of polystyrene MNPs in rats does not produce marked brain accumulation over three months.^1^ It does not, however, rule out differential chronic accumulation in the human brain, nor does it invalidate models in which long-term organ burdens are shaped primarily by slow retention in lipid-rich tissue rather than by vascular volume per se.

Resolving this issue will require experiments that explicitly bridge the present temporal and biological gap: chronic exposure designs, longer observation windows, polymer-resolved measurements, perfused tissues when feasible, and explicit separation between early distributional kinetics and true long-horizon retention. Until then, short-term rat biodistribution and chronic human bioaccumulation should not be treated as equivalent tests of the same hypothesis.
